# PrismaLuc luciferase enables tunable near-infrared bioluminescence for *in vivo* imaging

**DOI:** 10.1039/d6cb00175k

**Published:** 2026-09-07

**Authors:** Felipe Gama-Franceschi, Rachel Friedman Ohana, Chao Gao, Thomas Kirkland, Laura Mezzanotte

**Affiliations:** a Department of Radiology & Nuclear Medicine, Erasmus MC Cancer Institute, Erasmus University Medical Centre Rotterdam The Netherlands; b Department of Molecular Genetics, Erasmus MC Cancer Institute, Erasmus University Medical Centre Rotterdam The Netherlands; c Delft University of Technology, Delft The Netherlands; d Promega Corporation Madison Wisconsin USA

## Abstract

Bioluminescence imaging (BLI) enables non-invasive monitoring of biological processes in living animals, yet most bioluminescent reporters emit at fixed wavelengths with limited spectral flexibility, constraining their use in multiplexed and deep-tissue applications. Bioluminescence resonance energy transfer (BRET) offers a solution by coupling a luciferase to a proximal fluorescent protein or synthetic fluorophore that absorbs the emitted light and re-emits it at a longer wavelength. However, existing BRET systems built from luciferase–fluorescent protein fusions are limited by suboptimal energy transfer efficiency and the fixed photophysical properties of their genetically encoded chromophores. We recently introduced PrismaLuc, a high-efficiency BRET reporter, in which a circularly permuted NanoLuc is inserted into HaloTag, enabling efficient energy transfer to a covalently bound synthetic fluorophore whose emission wavelength is selected by choice of ligand. In our initial report, PrismaLuc was paired with JF549 HaloTag ligand for two-colour cell-based assays. Here, we extend PrismaLuc to *in vivo* bioluminescence imaging using far-red and near-infrared HaloTag ligands. In cultured cells, labelling with JF608 and JF669 HaloTag ligands produced emission peaks at 640 and 690 nm, respectively. Direct comparison with established BRET reporters revealed greater photon output at wavelengths above 665 nm, which are less attenuated in tissue. In tumour-bearing mice, PrismaLuc:JF669 produced an emission peak of approximately 700 nm, and *in vivo* fluorescence imaging confirmed sufficient JF669 HaloTag ligand bioavailability to label PrismaLuc expressed in xenograft tumours. Finally, dual-colour imaging in mice co-implanted with NanoLuc- and PrismaLuc-expressing cells demonstrated clear spectral separation, enabling multiplexed detection within the same animal. Together, these results establish PrismaLuc as a tunable platform for near-infrared and dual-colour bioluminescence imaging *in vivo*.

## Abstract 2

Bioluminescence imaging (BLI) enables non-invasive monitoring of biological processes in living animals, yet most bioluminescent reporters emit at fixed blue-yellow wavelengths, constraining their use in multiplexed and deep-tissue applications.

## Graphical abstract

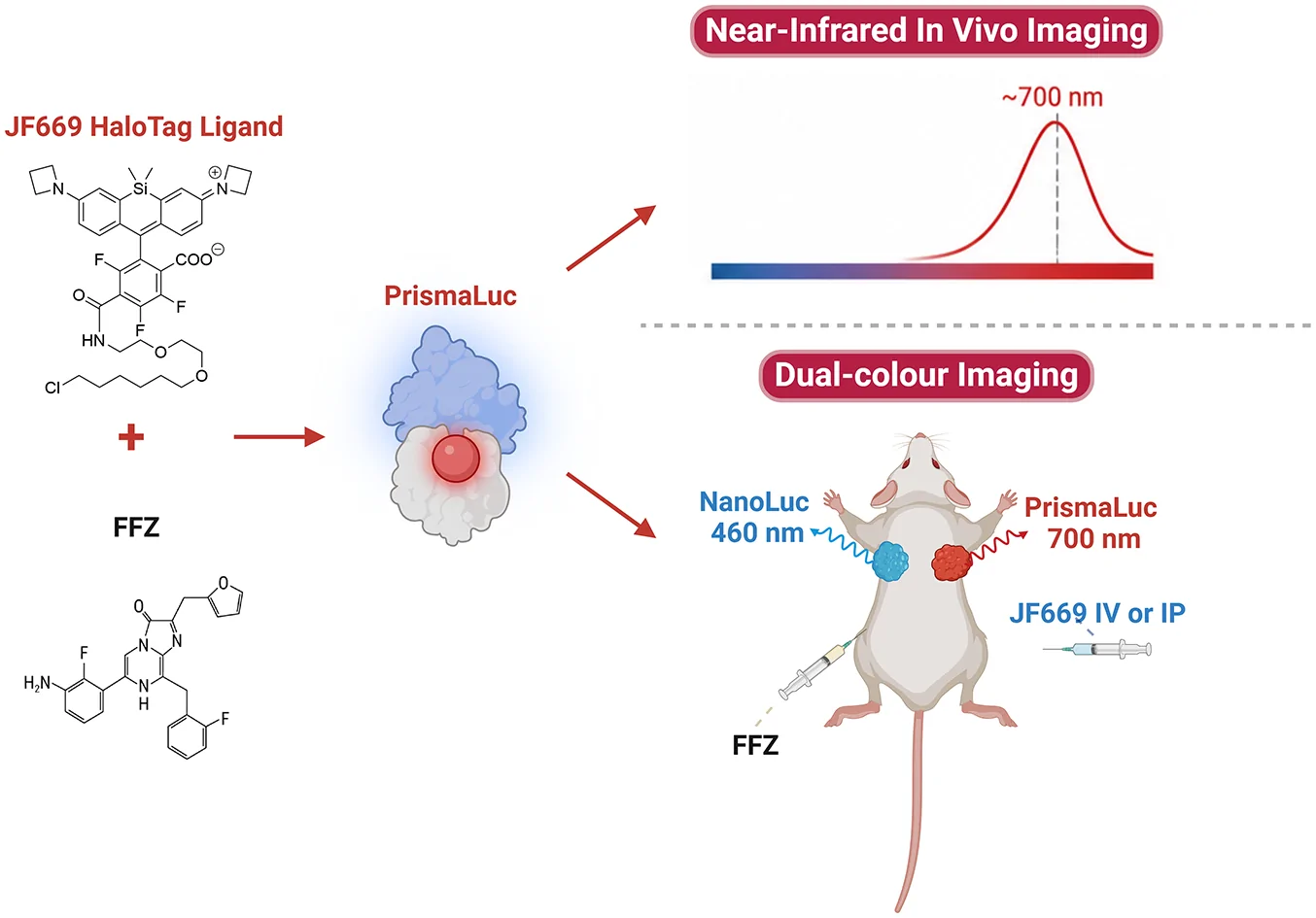

## Introduction

Bioluminescence imaging (BLI) is widely used for monitoring biological processes *in vitro* and *in vivo* owing to its high sensitivity, low background and independence of external excitation light. These features are particularly suited for studying dynamic biological processes across a wide range of experimental contexts, enabling non-invasive monitoring of cellular and molecular events in complex biological environments.^1,2^ However, *in vivo* applications impose specific demands that most existing reporters do not fully address. Bright luciferases such as NanoLuc, a small 19 kDa enzyme derived from Oplophorus gracilirostris,^3^ paired with fluorofurimazine (FFz),^4^ a substrate optimised for *in vivo* imaging, emit at fixed wavelengths in the blue-yellow range,^5^ which are strongly attenuated by tissue absorption and scattering.^6,7^ This limits detection sensitivity at depth and complicates multiplexed imaging in living animals. Overcoming these constraints requires reporters that combine bright bioluminescence with tunable, red-shifted emission.

To address these spectral limitations, several groups have turned to bioluminescence resonance energy transfer (BRET), which red-shifts luciferase emission by coupling it to a proximal fluorescent acceptor. Briefly, energy emitted from a luciferase donor is transferred to a nearby fluorescent acceptor and re-emitted at a longer wavelength.^8,9^ The efficiency of this process depends on the spectral overlap, proximity, and spatial orientation between donor and acceptor.^8,9^

Early BRET systems were commonly based on Renilla luciferase (RLuc),^8,10–12^ which demonstrated the feasibility of red-shifted BRET imaging *in vivo*, with RLuc–iRFP chimeras achieving near-infrared emission at 670–720 nm in mice.^13,14^ However, these systems were constrained by lower photon output, biliverdin-dependent chromophore maturation and fixed acceptor emission spectra. NanoLuc has since emerged as the preferred donor for *in vivo* BRET applications, owing to its small size, bright luminescence, narrow emission profile, improved spectral separation and compatibility with optimised imidazopyrazinone substrates.^3,4,9,15^

Among the NanoLuc-based BRET reporters developed to date, Antares and the red NanoLanterns have been used frequently for *in vivo* imaging due to their bright red emission. Antares fuses NanoLuc to two copies of CyOFP1,^16,17^ while the NanoLantern^12^ variants pair NanoLuc with fluorescent proteins such as mCherry^18^ or tdTomato.^19^ While these systems improve *in vivo* detection relative to unmodified NanoLuc, they rely on genetically encoded fluorescent protein acceptors whose emission spectra and photophysical properties are fixed at the time of construct design. An alternative to genetically encoded acceptors is the use of synthetic fluorophores as BRET acceptors introduced through chemogenetic tagging.

The self-labelling protein HaloTag covalently binds chloroalkane-tethered fluorophores, enabling interchangeable labelling with a broad range of synthetic dyes.^15,20^ The covalent and stoichiometric nature of HaloTag labelling further ensures quantitative occupancy while the ability to choose fluorophores from spectrally diverse families such as the Janelia Fluor (JF) dyes allows users to select ligands optimised for their experimental application. In particular, JF HaloTag ligands spanning the red to near-infrared spectral range extend emission into regions that are not well supported by fluorescent protein acceptors. These ligands combine favourable photophysical properties with demonstrated *in vivo* utility, including efficient labelling in mouse tissues,^21–23^ making them especially attractive as synthetic acceptors for BRET-based spectral tuning.^23–25^

In this context, we recently introduced PrismaLuc, a high-efficiency BRET reporter that integrates the strengths of NanoLuc brightness with the modularity of HaloTag labelling.^26^ The PrismaLuc architecture builds on the earlier work of Hiblot *et al.*,^27^ who first demonstrated that circularly permuted NanoLuc inserted into self-labelling tags enables BRET with covalently bound fluorophores. PrismaLuc retains the same circular permutation site but incorporates an optimised HaloTag insertion site, linker configuration, and stabilising mutations, achieving 88.9% apparent BRET efficiency compared with 75.7% for the Hiblot construct under matched biochemical conditions.^26^ In mammalian cells, PrismaLuc supports robust two-colour imaging, enabling simultaneous detection of blue NanoLuc and red-shifted PrismaLuc signals within the same system using a single substrate.^26^ In addition, ligand-dependent spectral tuning allows PrismaLuc to function both as a bioluminescent reporter and as a fluorescent tag, facilitating multimodal readouts including fluorescence-activated cell sorting. In this study, we extend the utility of PrismaLuc to *in vivo* near-infrared bioluminescence imaging. This requires systemic delivery of the HaloTag ligand, sufficient ligand bioavailability at the target tissue, and retention of BRET-shifted emission after ligand and substrate administration. Specifically, we evaluated PrismaLuc performance with JF HaloTag ligands spanning the visible to far-red range in both bacteria and mammalian cells, benchmarked its near-infrared photon output against established BRET reporters, examined ligand bioavailability following systemic administration, and demonstrated dual-colour bioluminescence imaging in tumour-bearing mice. Together, these results position PrismaLuc as a versatile, tunable high-performance bioluminescent platform for near-infrared and multiplexed imaging in both microbial systems and living animals.

## Results and discussion

### System-specific validation of PrismaLuc spectral tuning

**Fig. 1:**
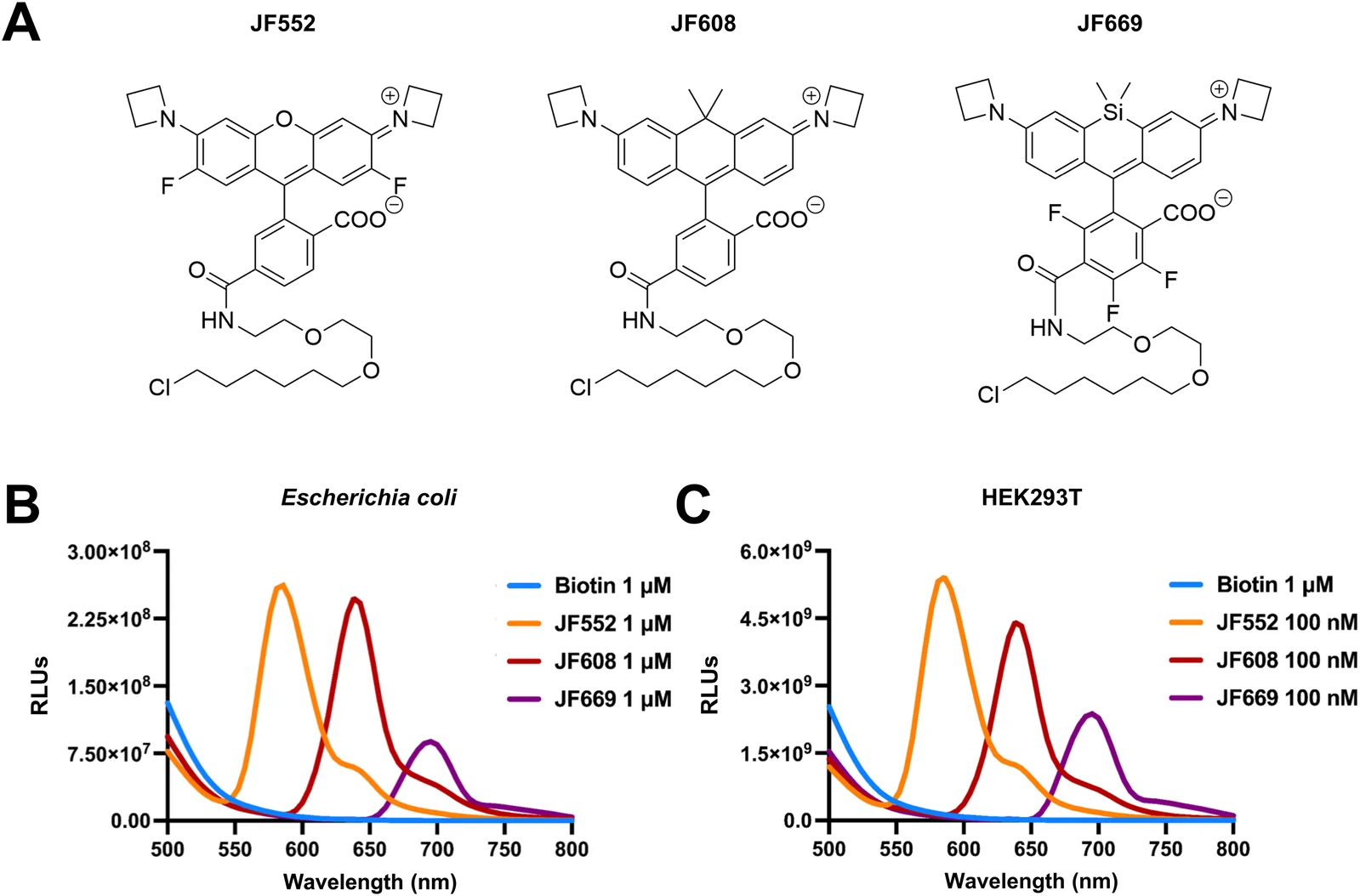
Validation of ligand-dependent spectral tuning of PrismaLuc using Janelia Fluor HaloTag ligands in bacterial and mammalian systems acquired on the IVIS. (A) Chemical structures of the JF HaloTag ligands used in this study: JF552, JF608, and JF669. Emission spectra for *E. coli* (B) and HEK293T (C) expressing PrismaLuc labelled with biotin, JF552, JF608, or JF669 HaloTag ligands following addition of Nano-Glo substrate. PrismaLuc exhibited ligand-dependent, red-shifted emission with peaks at approximately 580 nm (JF552), 640 nm (JF608), and 690 nm (JF669), while biotin controls showed blue emission, as expected. Spectra represent the mean of quadruplicate wells; SD is omitted for clarity.

PrismaLuc functionality and spectral tuning have previously been demonstrated in mammalian cells by Porter *et al.*^26^ Here, we first confirmed that ligand-dependent spectral tuning is broadly maintained across biological systems, including bacterial and additional mammalian cell lines using the IVIS-based spectral acquisition and labelling conditions employed in subsequent *in vivo* experiments. PrismaLuc was cloned into a bacterial expression vector and expressed in *E. coli.* For mammalian expression, stable HEK293T and HCT116 cell lines were generated *via* lentiviral transduction and enriched by fluorescence-activated cell sorting (FACS). Upon labelling with HaloTag ligands JF552,^28^ JF608^23^ or JF669^25^ (Fig. 1A), followed by addition of Nano-Glo substrate, PrismaLuc produced bright bioluminescent signals with distinct emission peaks centred at approximately 580 nm, 640 nm, and 690 nm, respectively, whereas biotin controls exhibited an emission centred in the blue spectrum (Fig. 1B and C). Because IVIS spectral acquisition was limited by the available emission filters and began at 500 nm, the 460 nm NanoLuc donor peak was not fully captured in these measurements. These results confirm efficient bioluminescence resonance energy transfer (BRET) between the circularly permuted NanoLuc and the covalently bound fluorophores and demonstrate that PrismaLuc enables ligand-dependent modulation of emission wavelength using a single genetic construct. Because the bacterial and mammalian assays used different cell numbers, expression systems, and labelling conditions, as well as differences in ligand accessibility (cell membrane *vs.* Gram-negative envelope), absolute signal intensities are not directly comparable between host systems. Nevertheless, the relative emission intensities across ligands largely reflect the expected correlation between BRET efficiency and the degree of spectral overlap between donor emission and acceptor excitation. The consistency of spectral tuning across prokaryotic and mammalian systems, combined with the ability to isolate labelled cells based on fluorescence, highlights the versatility of PrismaLuc as a multifunctional reporter.

### PrismaLuc exhibits enhanced near-infrared output compared to established BRET reporters

**Fig. 2:**
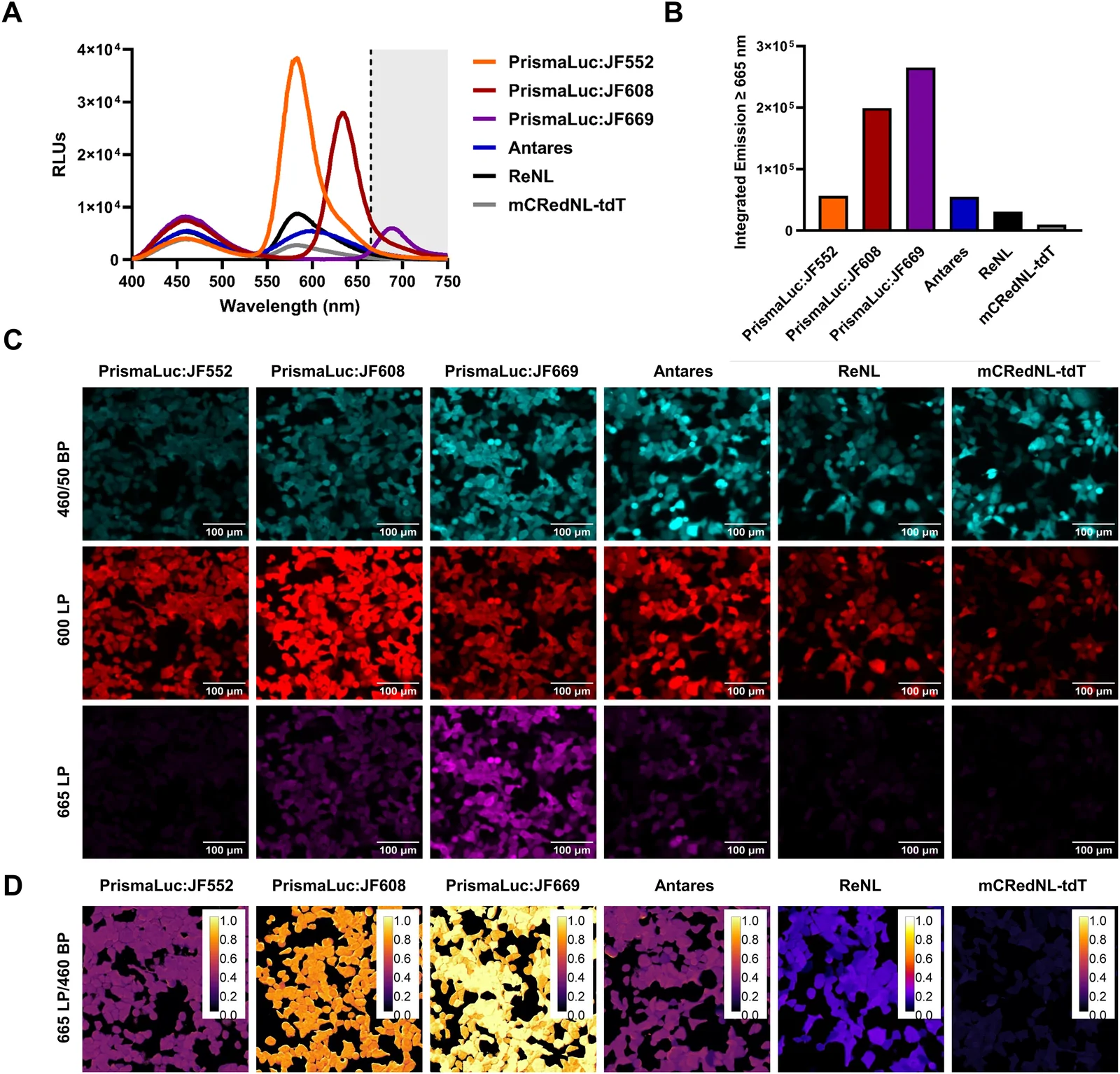
Spectral comparison of PrismaLuc and established BRET systems in HCT116 cells. (A) Raw emission spectra of HCT116 cells expressing PrismaLuc, Antares, or NanoLantern following mRNA transfection and substrate addition. The shaded region indicates wavelengths above 665 nm. (B) Integrated photon emission above 665 nm for each reporter construct, highlighting enhanced far-red photon output for PrismaLuc labelled with JF608 and JF669 HaloTag ligands. (C) Representative bioluminescence microscopy images acquired using the Galaxy imaging system equipped with 460/50 BP, 600 LP, and 665 LP filters. All images were captured with 1.5 min exposures and displayed with a common intensity scale. (D) Per-pixel ratiometric images (665 LP/460 BP) generated from galaxy imager data. A cell mask was applied to both channels prior to division to exclude background regions. Higher values indicate more complete spectral shift to the far-red acceptor. A common calibration bar is applied across all panels.

In preparation for our *in vivo* analysis, we performed a direct comparison of PrismaLuc bound to red, far-red and near-infrared HaloTag ligands with Antares^16,17^ and NanoLanterns ReNL^19^ and mCRedNL-tdT^18^ which use fluorescent proteins as acceptors. All constructs were expressed in HCT116 cells *via* mRNA transfection at equal molar amounts to reduce expression variability. FFz was used as the substrate, consistent with its intended use in subsequent *in vivo* experiments. Following expression, PrismaLuc-expressing cells were labelled with HaloTag ligands JF552, JF608, or JF669. Spectral analysis following FFz substrate addition revealed that PrismaLuc generated robust bioluminescent signals with tunable emission peaks spanning the visible to far-red range, depending on the ligand used, while Antares and the NanoLanterns displayed fixed emission profiles (Fig. 2A). Normalisation to the 460 nm donor peak revealed relative BRET efficiency for each reporter independently of expression level, confirming that differences in red-shifted emission reflect intrinsic energy transfer properties rather than variation in reporter abundance (Fig. S1A). To quantify performance in the spectral region most relevant for *in vivo* imaging, integrated photon emission at wavelengths above 665 nm (Fig. 2B) was extracted from the emission scans. PrismaLuc:JF669 produced integrated photon output 1.3-fold higher than PrismaLuc:JF608, 4.7-fold higher than Antares, 8.6-fold higher than NanoLantern ReNL, and 26-fold higher than NanoLantern mCRedNL-tdT (Fig. 2B). PrismaLuc:JF552 showed 4- to 5-fold lower signal than JF608 and JF669 in this spectral window, consistent with its shorter emission maximum (Fig. 2B). To confirm these spectral differences under imaging conditions relevant to *in vivo* applications, cells were imaged using the GloMax Galaxy Bioluminescence Imager System (Promega) equipped with 460/50 nm bandpass (BP), 600 nm long-pass (LP), and 665 nm long-pass filters (Fig. 2C). All reporters produced detectable signal in the donor-associated 460/50 nm BP filter. Using the 600 nm LP filter, all BRET reporters produced acceptor signal, with PrismaLuc:JF608 showing the brightest emission. Critically, with the 665 nm LP filter, the spectral window most relevant for *in vivo* detection conditions, PrismaLuc:JF669 produced the strongest signal, PrismaLuc:JF608 and Antares were both markedly weaker and NanoLantern variants were undetectable. To complement the integrated spectral analysis, we generated ratiometric images by dividing the 665 LP signal by the 460 BP signal on a per-pixel basis (Fig. 2D). This ratio visualises the extent of the spectral shift for each reporter, with higher values indicating more complete energy transfer to the far-red acceptor. PrismaLuc:JF669 showed the highest acceptor-to-donor ratio across the imaging field, in good agreement with the integrated spectral analysis at ≥ 665 nm, confirming that per-pixel ratiometric imaging provides a reliable spatial readout of the BRET spectral shift. Antares and the NanoLantern variants showed low ratios, reflecting limited emission in the far-red relative to their donor signal (Fig. 2D). This result, together with the integrated spectral analysis, provided the rationale for selecting the JF669 HaloTag ligand for subsequent *in vivo* experiments.

### Systemic administration of HaloTag ligands enables *in vivo* bioavailability

**Fig. 3:**
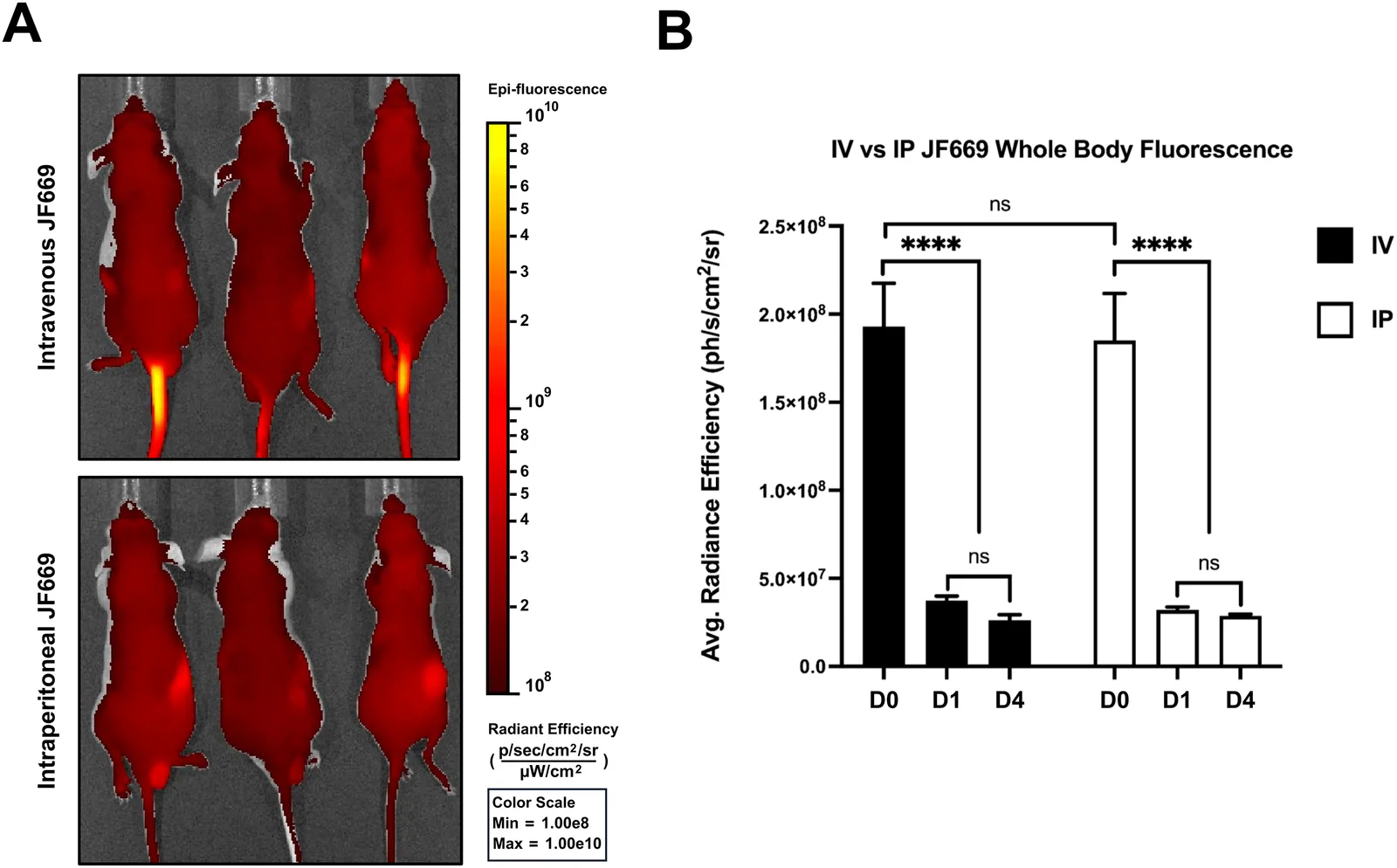
*In vivo* bioavailability and clearance of JF669 HaloTag ligand following systemic administration. (A) Whole-body fluorescence imaging of BALB/c nude mice bearing HCT116 tumours following intraperitoneal or intravenous administration of JF669 HaloTag ligand (2.5 nmol). Fluorescence signal was detected 1 minute after injection, confirming systemic ligand distribution and bioavailability. (B) Quantification of fluorescence signal in whole-body regions of interest (ROIs) over time following IV and IP administration. Fluorescence intensity decreased substantially at 24 and 96 hours post-injection, indicating progressive clearance of the ligand.

**Fig. 4:**
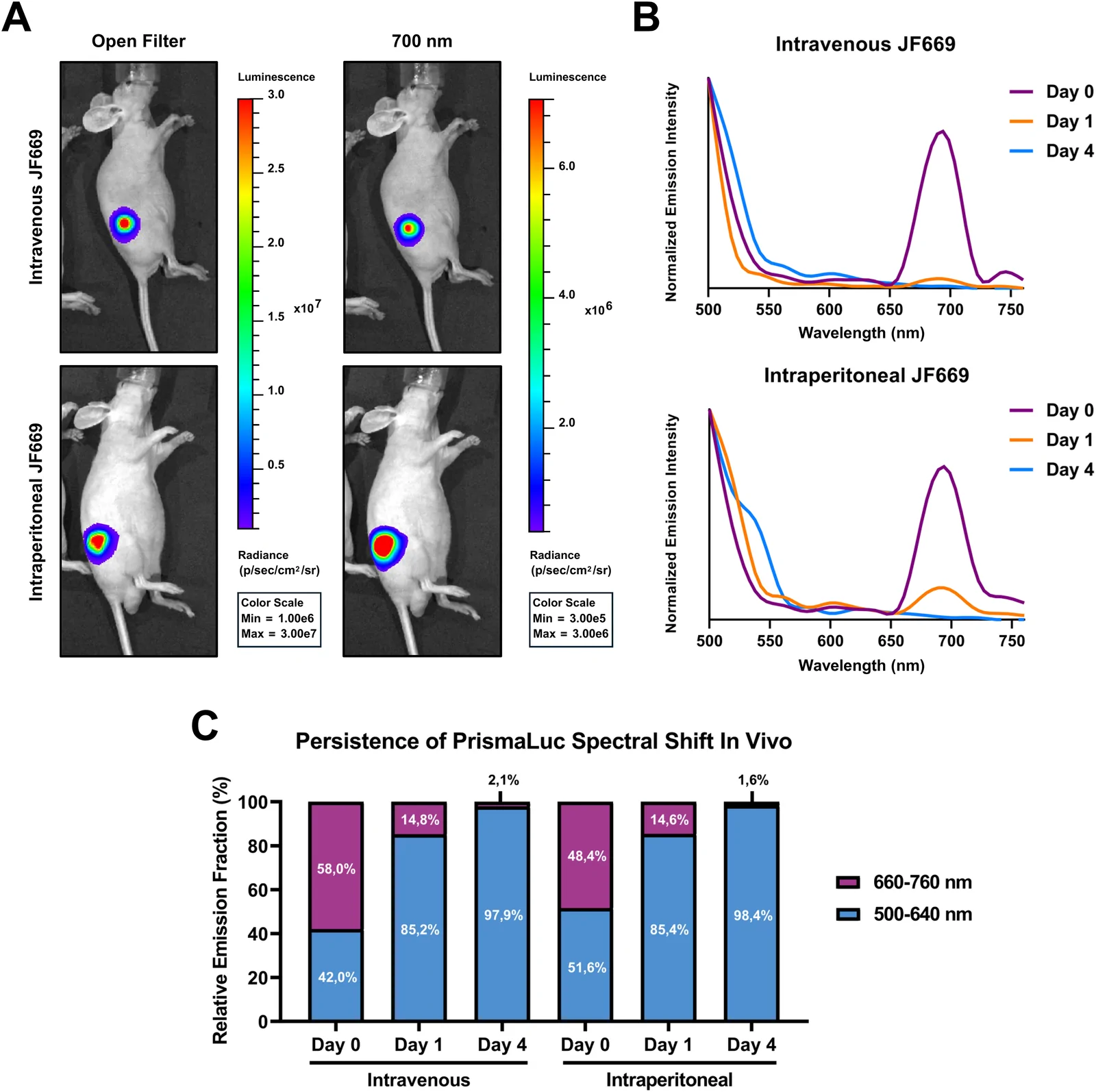
*In vivo* bioluminescence imaging of PrismaLuc-labelled HCT116 tumours following JF669 HaloTag ligand administration. (A) Bioluminescence images of mice bearing HCT116 PrismaLuc tumours following IV (top) or IP (bottom) administration of JF669 HaloTag ligand and FFz substrate acquired with open and 700 nm bandpass filters. (B) *In vivo* normalised emission spectra of HCT116 tumours expressing PrismaLuc following intravenous or intraperitoneal administration of JF669 HaloTag ligand (2.5 nmol), with bioluminescence measured after FFz substrate injection at day 0, day 1, and day 4. Spectral analysis revealed red-shifted emission with a peak at approximately 700 nm. Signal intensity decreased over time, reflecting turnover of labelled PrismaLuc and inability to label newly synthesized reporter due to ligand clearance. (C) Relative contributions of shorter-wavelength emission from 500–640 nm and near-infrared emission from 660–760 nm to the total spectrally resolved tumour signal measured from 500–760 nm. The contribution of each wavelength range was calculated by dividing the summed signal within that range by the summed signal across all filters from 500–760 nm. Percentages are shown within or above the bars.

**Fig. 5:**
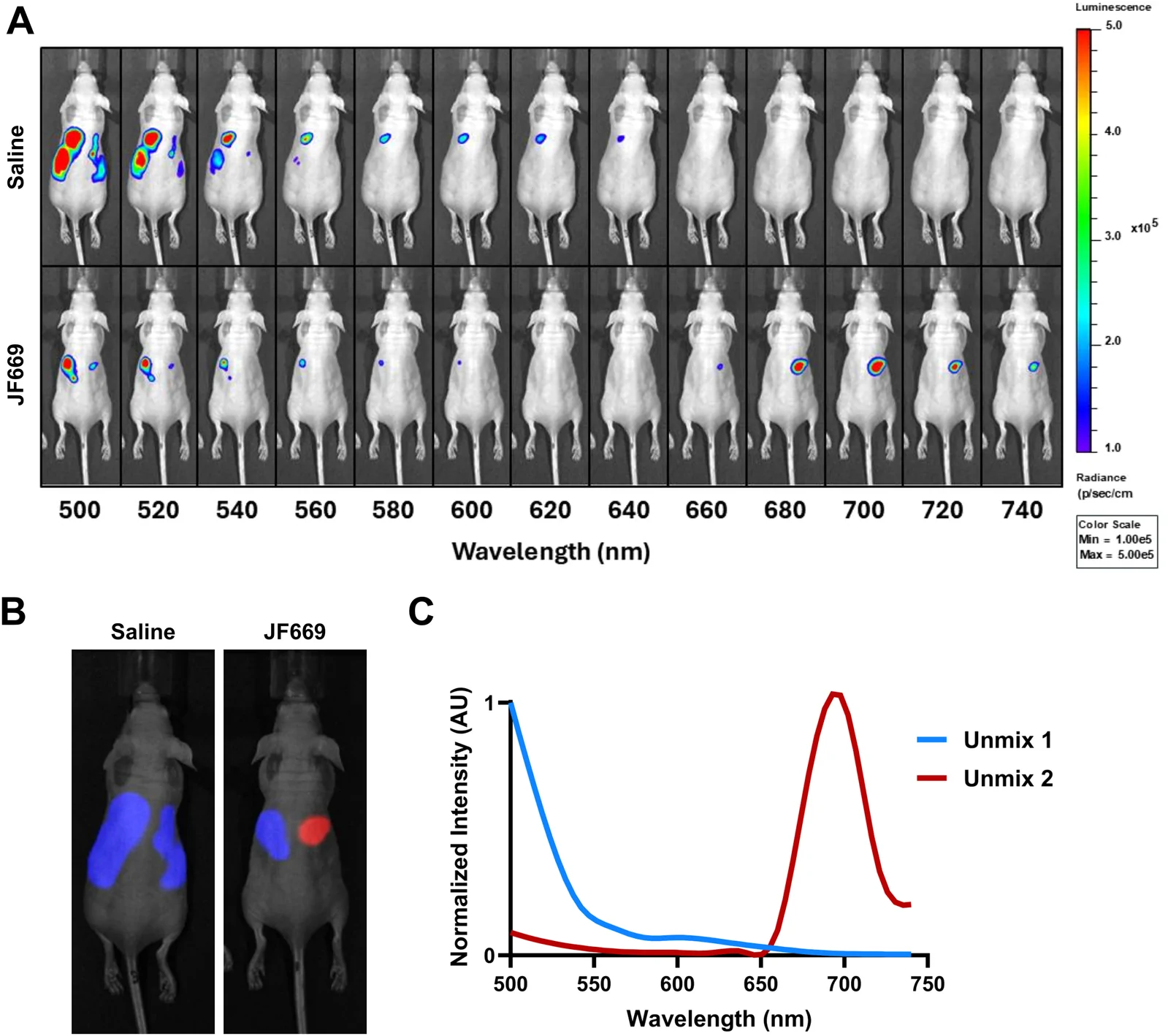
Dual-colour *in vivo* imaging of NanoLuc and PrismaLuc in tumour models. (A) Bioluminescence images of BALB/c nude mice bearing HEK293T cells expressing NanoLuc (left flank) and PrismaLuc (right flank) following FFz substrate administration. Saline-treated mice showed predominantly blue emission from both sites, whereas JF669 HaloTag ligand-treated mice showed near-infrared emission selectively from the PrismaLuc-expressing site. (B and C) Spectral unmixing of bioluminescence signals acquired using sequential band-pass filters and analysed using the library-guided spectral unmixing of Living Image software demonstrates clear separation of donor-dominant and acceptor-dominant emission components within the same animal.

A prerequisite for *in vivo* application of PrismaLuc is that the HaloTag ligand reaches the target tissue at sufficient concentration to label the reporter. Far-red Janelia Fluor HaloTag ligands, including JF669, have previously been validated for *in vivo* HaloTag labelling in mice, particularly in neuroimaging applications. JF669-HaloTag ligand has been delivered by retro-orbital injection at doses of 100–200 nmol to label HaloTag-expressing tissues in the mouse brain.^21,22^ Both studies also identified JF552 and JF669 ligands as among the most bioavailable HaloTag ligands for *in vivo* labelling.^21,22^ However, these studies focused on CNS or retro-orbital delivery, whereas whole-body ligand bioavailability for tumour bioluminescence imaging remains less explored. To assess the *in vivo* bioavailability of JF669 HaloTag ligand in this context, BALB/c nude mice bearing HCT116 tumours were administered JF669 HaloTag ligand *via* either intraperitoneal (IP) or intravenous (IV) injection at substantially lower doses of 2.5, 5, or 10 nmol. Fluorescence imaging showed that ligand administration was well tolerated up to 10 nmol (Fig. 3–5 and Fig. S2). Among the doses tested, 2.5 nmol was sufficient to generate a detectable PrismaLuc BRET signal. Signal was quantified for mice receiving 2.5 nmol JF669 HaloTag ligand by IP or IV administration, confirming systemic ligand distribution and tumour bioavailability (Fig. 3A). Over time, fluorescence intensity decreased substantially, with a 10- to 20-fold reduction observed at 24 and 96 hours post-injection, indicating progressive clearance of the ligand (Fig. 3B).

### PrismaLuc enables near-infrared bioluminescence imaging *in vivo*

To evaluate the effect of ligand delivery route on the BRET-shifted bioluminescence signal, mice bearing HCT116 tumours stably expressing PrismaLuc were injected with JF669 HaloTag ligand, followed by administration of FFz substrate. Bioluminescence imaging was performed using an IVIS Spectrum system (Revvity) with an open emission filter and sequential band-pass filters spanning 500–760 nm (Fig. 4). Red-shifted emission was detected at the tumour site, with an *in vivo* emission peak at approximately 700 nm (Fig. 4B), consistent with the 690 nm peak observed in cultured cells. This corresponds to a ∼240 nm red-shift relative to unmodified NanoLuc, which is consistent with efficient BRET in living animals. This emission falls within a spectral range that has been exploited by several red-shifted bioluminescence systems to improve *in vivo* sensitivity. For example, substrate-engineered approaches such as AkaLumine–HCl/AkaLuc (∼650 nm) and AkaSuke/DkumLuc1 (∼680 nm),^29–31^ infraluciferin-based systems (∼705 nm),^32^ and red-shifted click beetle luciferases CBR2/NH_2_–NpLH_2_ (∼730 nm)^33,34^ have each extended emission into the far-red and near-infrared range. NanoLuc-based BRET reporters such as Antares (∼590 nm) and NirNL (∼680 nm) demonstrate a shifted emission through energy transfer to fluorescent acceptors CyOFP1^16,17^ and NirFAP680,^35^ respectively. Accordingly, PrismaLuc:JF669, with its ∼700 nm peak, ranks among the most red-shifted NanoLuc-based reporters available.

Representative open filter and 700 nm images demonstrate that near-infrared signal is detected on day 0 (Fig. 4A). Full spectral image series from representative animals following IV and IP ligand administration at day 0, day 1 and day 4 are provided in Fig. S3. Normalisation of the tumour-associated signal at each wavelength to the corresponding 500 nm measurement revealed a pronounced near-infrared emission component centred around 680–700 nm at the earliest post-administration time point (Fig. 4B).

To further quantify the spectral distribution, the measured emission was divided into two fractions spanning the IVIS filters: a shorter-wavelength (500–640 nm) and a near-infrared fraction (660–760 nm) (Fig. 4C). At the earliest measured time point, a substantial proportion of the spectrally resolved tumour signal was detected within the near-infrared range: 58% following IV and 48.4% following IP ligand administration, demonstrating that either route can provide sufficient ligand exposure at the tumour by 45 minutes post injection. The far-red contribution decreased over time to ∼15% at day 1 for both administration routes, indicating that a residual PrismaLuc:JF669 spectral shift remained detectable at 24 h, although substantially reduced. By day 4, the 660–760 nm fraction had declined to ∼2%, which likely reflects the tail of the native NanoLuc emission rather than a persistent BRET (Fig. 4B and C). As these time points were selected for an initial proof-of-concept study, we did not assess whether a stronger far-red contribution occurs at intermediate intervals. Future studies should examine additional time points within the first 24 h to determine the optimal post-injection imaging window.

Because the labelling of PrismaLuc is covalent, the loss of BRET signal likely reflects two converging processes: turnover of PrismaLuc which was already labelled, and inability to label newly synthesized PrismaLuc due to clearance of the free ligand from the circulation. This interpretation is consistent with the ligand clearance kinetics observed by fluorescence imaging (Fig. 3B) and suggests that re-administration of the HaloTag ligand prior to imaging sessions may be necessary to maintain optimal labelling in longitudinal studies.

### PrismaLuc supports dual-colour bioluminescence imaging using a single substrate

To evaluate whether PrismaLuc enables dual-colour imaging *in vivo*, HEK293T cells expressing NanoLuc (left flank) or PrismaLuc (right flank) were subcutaneously implanted into BALB/c nude mice. One group was pre-treated with 10 nmol JF669 HaloTag ligand, whereas the control group received saline in place of the ligand. All mice received FFz substrate and were imaged 15 minutes later using an IVIS Spectrum system (Revvity). In saline-treated mice, both flanks produced predominantly blue emission, consistent with the ∼460 nm emission of both native NanoLuc and unlabelled PrismaLuc (Fig. 5A–C). Following JF669 HaloTag ligand administration, near-infrared emission was observed selectively at the PrismaLuc site, while the NanoLuc site retained its blue emission profile. This generated a clear spectral separation of >240 nm between NanoLuc emission (∼460 nm) and PrismaLuc:JF669 emission (∼700 nm), supporting software-assisted spectral unmixing of the two spatially separated signals within the same animal (Fig. 5C).

These data provide a proof-of-concept demonstration that spatially separated NanoLuc- and PrismaLuc-expressing cell populations can be distinguished *in vivo* using a single substrate, eliminating the need for sequential substrate administration. This provides practical advantages over conventional dual-luciferase approaches that rely on separate enzyme–substrate pairs, where differences in substrate pharmacokinetics, biodistribution, delivery efficiency, and stability can introduce variability between imaging channels.^34,36,37^ In contrast, the NanoLuc/PrismaLuc:JF669 pairing allows both signals to be acquired following administration of the same substrate, supporting simultaneous imaging, software-assisted spectral unmixing, and improved temporal comparability between reporter populations. Compared with systems based solely on spectrally distinct luciferases, PrismaLuc further expands multiplexing flexibility by allowing the emission wavelength to be tuned through ligand choice rather than reporter re-engineering.

A trade-off of this ligand-tunable strategy is that optimal red-shifted emission requires a separate HaloTag ligand administration step and sufficient reporter occupancy to maximise BRET efficiency. Incomplete labelling may result in residual blue donor emission, which could complicate spectral unmixing in some experimental settings. However, because HaloTag labelling is covalent, labelled reporter molecules remain labelled once the ligand has bound. Therefore, optimal performance depends primarily on ligand bioavailability, dose, and timing, parameters that can be optimised according to the imaging application.

The present experiment represents a favourable configuration, as the two cell populations, each expressing a different reporter, were spatially separated, allowing the unmixed signals to be visually verified by their distinct locations. The approach is therefore best suited for applications involving spatially or anatomically separated reporter sources, such as distinct tumours, lesions, or experimental and reference sites within the same animal. Applications requiring spectral unmixing of co-localised reporters would require additional validation using defined reporter ratios and appropriate single-colour controls.

## Conclusions

PrismaLuc establishes a modular strategy for tunable bioluminescence imaging by combining NanoLuc brightness with HaloTag ligand flexibility. Through interchangeable synthetic fluorophores, PrismaLuc enables emission tuning across the visible to near-infrared spectrum without requiring modification of the underlying genetic construct, providing a practical route to adapt reporter emission to tissue depth, spectral separation or instrument configuration. While near-infrared BRET has previously been demonstrated *in vivo* using RLuc–iRFP chimeras,^13,14^ PrismaLuc achieves comparable emission wavelengths with a brighter donor and the added advantage of spectral tunability through ligand choice.

Beyond tumour imaging, PrismaLuc could support a range of applications in which non-invasive, multiplexed or deep-tissue optical readouts are needed. Its compatibility with bacterial expression suggests potential use for tracking engineered microbes, probiotic colonisation, or bacterial biodistribution *in vivo*, and ongoing work is exploring the application of PrismaLuc for imaging bacterial systems in the gut. In mammalian systems, the ability to spectrally separate NanoLuc and PrismaLuc signals using a single substrate could enable simultaneous monitoring of spatially or anatomically separated cell populations, or multiplexed assessment of cell therapies together with disease burden. The ligand-tunable nature of the system may also allow users to select fluorophores that best match anatomical depth or experimental requirements.

Together, these properties position PrismaLuc as a versatile platform for multiplexed and near-infrared bioluminescence imaging, with possible future applications in microbial imaging, immune-cell tracking, cell therapy monitoring, and complex *in vivo* models requiring simultaneous detection of multiple biological processes. The present study establishes proof of concept in subcutaneous, spatially separated tumour models. Further studies will be needed to define optimal dosing and imaging timepoints and to evaluate spectral discrimination in deeper or overlapping biological settings.

## Materials and methods

### Cloning of bacterial expression vectors and transformation

To generate bacterial expression constructs encoding PrismaLuc, the PrismaLuc™ coding sequence^26^ was subcloned into our previously developed plasmid JCrT1^38^ using Gibson Assembly, replacing the CBG2 reporter gene. The vector backbone, containing the kanamycin resistance gene, plasmid origin of replication, constitutive J23119 promoter, and N-terminal 6× His tag, was amplified from JCrT1.

For Gibson assembly, all fragments were designed with 40 to 42 base pairs homology arms. PCR amplification, assembly reaction and bacterial transformation were performed as described in the supplementary section of Maranesi *et al.*, 2025.^38^ Primer sequences used for cloning are listed in Table S1. Following assembly, resulting plasmids were transformed into electrocompetent *E. coli* Seattle 1946 (ATCC25922), using the same protocol as described in the SI section of Maranesi *et al.*, 2025.^38^

### Bacterial *in vitro* bioluminescence measurements

*Escherichia coli* expressing PrismaLuc were grown overnight (∼15 h) in LB medium at 37 °C with shaking at 170 rpm. PrismaLuc™-expressing cultures were supplemented with JF552, JF608, JF669, or biotin HaloTag® ligands (Promega) at a final concentration of 1 µM. Cultures were centrifuged (10 min, 7000 rpm), washed twice with PBS, and resuspended at 1 × 10^7^ cells in 100 µL PBS containing 250-fold dilution of the Nano-Glo® substrate (Promega). Luminescence was measured 10 min after substrate addition using an IVIS imager (Revvity) at room temperature. Emission spectra were acquired using sequential 20 nm band-pass filters from 500 to 800 nm, reflecting the available IVIS filter set. Spectral measurements were performed in quadruplicate wells within the same plate.

### Cell culture of HEK293T and HCT116

Human Embryonic Kidney 293T cells (HEK293T) (ATCC CRL-3216) and Human Colorectal Tumour 116 cells (HCT116) (ATCC CCL-247) were used in this study. Both cell lines were cultured in Dulbecco's modified Eagle's medium (DMEM), pH 7.4 (Sigma-Aldrich), supplemented with 10% fetal bovine serum (FBS) and 1% penicillin/streptomycin. The cells were maintained in 37 °C humidified incubators with a 5% CO_2_ atmosphere. Passaging was performed using either 75 cm^2^ tissue culture flasks (Sarstedt) or 10 cm cell culture dishes (Corning). Additionally, the cells were tested for mycoplasma contamination.

### Viral production

Lentiviral particles were generated by transiently transfecting HEK293T packaging cells with a lentiviral plasmid (either pCDH-EF1-NanoLuc-T2A-copGFP or pCDH-EF1-PrismaLuc) and the three packaging plasmids pCMV-VSV-G (Addgene #8454), pMDLg/pRRE (Addgene #12251), and pRSV-Rev (Addgene #12253) using PEI transfection reagent at 1 mg mL^−1^ per 1 µg of DNA. Lentiviral supernatants were collected at 48 and 72 hours post-transfection, filtered through a 0.45 µm membrane, and quantified using an antigen-capture HIV p24 ELISA kit (ZeptoMetrix).

### Transduction

HEK293T and HCT116 cells were grown in culture dishes until reaching about 70% confluence in culture medium as described above. Cells were then infected with the appropriate lentiviral stock, leading to the co-expression of NanoLuc–CopGFP or expression of PrismaLuc. For transduction, pseudoviral particles were added at a multiplicity of infection (MOI) of 1 after addition of polybrene infection/transfection reagent (Sigma-Aldrich) at a final concentration of 8 µg mL^−1^. Following infection, cells were maintained in appropriate culture conditions to ensure proper integration of the genes and consequent expression of the desired proteins.

### Sorting protocol

To obtain homogeneous cell populations, PrismaLuc™-expressing cells were incubated for 2 hours in Opti-MEM without phenol red (ThermoFisher) with 2% FBS in the presence of 100 nM JF552 HaloTag® ligand in a total volume of 200 µL. In parallel, NanoLuc®–CopGFP-expressing cells and the PrismaLuc™ cells were washed once with Opti-MEM™ without phenol red and subsequently trypsinized. Detached cells were then sorted using FACS on a BD FACSAria III (BD Biosciences). Fluorescence filters used for sorting included 585/15 for JF552 and 515/20 for CopGFP.

### HEK293T and HCT116 *in vitro* bioluminescence measurements

Sorted HEK293T–PrismaLuc™ and HCT116–PrismaLuc™ cell lines were cultured in Opti-MEM without phenol red (ThermoFisher) supplemented with 2% FBS. Cells were seeded into sterile, black flat-bottom 96-well plates (Greiner) at a seeding density of 20 000 cells per well. 24 hours after seeding, the JF552, JF608 and JF669 HaloTag® ligands (Promega) were added to the wells to a final concentration of 100 nM, while 1 µM Biotin HaloTag® ligand (Promega) was used as a control. After two hours of ligand incubation, cultures were washed twice by replacing the supernatant to remove excess ligand with PBS. For the bioluminescence assay, 100 µL of Opti-MEM containing 250-fold dilution of the Nano-Glo® substrate (Promega) was added to each well containing cells. Luminescence was measured 10 minutes after substrate addition using the IVIS imager (Revvity) at room temperature. Photon flux was measured in quadruplicates using sequential 20 nm band-pass filters ranging from 500 nm to 800 nm, reflecting the available IVIS filter set, to calculate the emission spectra.

### HCT116 mRNA transfection and labelling with JF HaloTag Ligands

To compare the performance of PrismaLuc with established, red-shifted BRET systems, we performed a side-by-side benchmarking assay in HCT116 cells using mRNA transfection. The design of constructs encoding PrismaLuc,^26^ Antares,^16,17^ and NanoLanterns (mCred NL-tdT and ReNL)^18,19^ was based on published sequences. Linear DNA templates suitable for *in vitro* transcription were synthesized (Elegen), and mRNAs were produced using RiboMAX™ Large Scale RNA Production System (Promega) with incorporation of AG CleanCap (TriLink; N-7113) and modified nucleotide (5′-methoxy UTP; N-1093) to enhance stability and translation efficiency. Following DNase treatment, mRNAs were purified by lithium chloride precipitation (ThermoFisher; AM9480) according to the manufacturer's instructions.

HCT116 cells were seeded in white 96-well plates at a density of 1 × 10^5^ cells per well in Opti-MEM (phenol-red free; Gibco) supplemented with 2% fetal bovine serum (Gibco) and penicillin–streptomycin (Gibco) and incubated at 37 °C with 5% CO_2_ for 18–24 hours. Equal molar amounts of mRNA were transfected using ViaScript® mRNA transfection reagent (Promega) at a 1 : 5 mRNA-to-reagent ratio, following the manufacturer's protocol. Cells were incubated for 18–24 hours post-transfection to allow protein expression.

For PrismaLuc labelling, cells were treated 4 hours post-transfection with HaloTag® ligands (JF552, JF608, or JF669, Promega) at a final concentration of 100 nM and incubated overnight to allow covalent labelling. No wash protocol was applied prior to measurement. For bioluminescence measurements, FFz substrate (Promega) was diluted in Opti-MEM and added to each well to a final 435-fold dilution. Following 1 minute of mixing, emission spectra were acquired using a Tecan Infinite M1000 PRO plate reader with monochromator-based emission scanning at 1 nm intervals. Emission profiles were normalised to the 460 nm donor peak to allow comparison between constructs.

### Mice strains and tumour cell inoculation

BALB/c nude mice (6–8 weeks old) were obtained from Charles River Laboratory and housed in the animal facility at Erasmus MC, Rotterdam, The Netherlands. All mice were provided access to food and water *ad libitum*. Mice were subcutaneously injected bilaterally under general anaesthesia (2.5% isoflurane) with 50 µL of 5 × 10^5^ HEK293T or HCT116 cells in PBS (each side with either NanoLuc® or PrismaLuc™ cell lines). HCT116 tumour growth was assessed until they were visualizable and reached a palpable size.

### *In vivo* bioavailability imaging of JF669 HaloTag ligand

BALB/c nude mice bearing subcutaneous HCT116 xenograft tumours were used for *in vivo* fluorescence biodistribution studies. JF669 HaloTag® ligand was administered at a dose of 2.5 nmol per mouse *via* either intravenous (IV) or intraperitoneal (IP) injection. Saline-injected mice served as controls. Mice were anaesthetized with 2% isoflurane and imaged using an IVIS Spectrum imaging system (Revvity). Whole-body fluorescence images were acquired approximately 1 min and 24 h post-injection using an excitation wavelength of 680 nm and an emission wavelength of 720 nm. Fluorescence signal was quantified using Living Image software by drawing regions of interest (ROIs) over tumours and major anatomical regions. Data were reported as radiant efficiency for comparison of biodistribution and clearance between administration routes.

### *In vivo* bioluminescence imaging

*In vivo* bioluminescence imaging was performed in mice bearing HCT116 tumours or in mice injected with HEK293T cells expressing PrismaLuc. For HCT116 tumour imaging, mice received a single injection of JF669 HaloTag® ligand (2.5 nmol per mouse) on day 0. Fifteen minutes later, FFz substrate was administered intraperitoneally (1.3 µmol in 100 µL PBS). For HEK293T cell experiments, mice received 10 nmol JF669 HaloTag® ligand, followed by FFz substrate injection 30 min later. Bioluminescence images were acquired 15 min after FFz substrate administration using an IVIS Spectrum system under 2% isoflurane anaesthesia. Images were collected using an open emission filter and sequential 20 nm band-pass filters spanning 500–800 nm, with an *f*/1 aperture, 30 seconds exposure time per filter, and FOV C. Longitudinal imaging of HCT116 tumour-bearing mice was performed on days 0, 1, and 4 following a single JF669 HaloTag® ligand injection on day 0, with FFz substrate administered prior to each imaging session. HEK293T cell imaging was performed on day 0 only. Bioluminescence signal was quantified as total photon flux (photons s^−1^) within ROIs drawn over tumours or cell injection sites. Spectral unmixing was performed using Living Image software using the library-guided function as described by Zambito & Mezzanotte^34^ to distinguish donor-dominant and far-red acceptor emission components where indicated.

### Safety and animal guidelines

All lentiviral work was conducted in a biosafety level II facility in accordance with national guidelines for genetically modified organisms and approved by the Erasmus Medical Centre Biosafety Committee. All animal experiments were approved by the Erasmus MC Bioethics Committee and performed in compliance with the Dutch Experiments on Animals Act (WoD) and European Directive 2010/63/EU under the national CCD license AVD101002017867.

## Author contributions

Felipe Gama-Franceschi: conceptualization, methodology, investigation, validation, formal analysis, data curation, visualization, writing – original draft and writing – review & editing. Rachel Friedman Ohana: conceptualization, investigation, formal analysis, resources, and writing – review & editing. Chao Gao: resources, methodology, and writing – review & editing. Thomas Kirkland: conceptualization, resources, methodology, and writing – review & editing. Laura Mezzanotte: conceptualization, methodology, investigation, supervision, project administration, funding acquisition, and writing – review & editing.

## Conflicts of interest

Rachel Friedman Ohana, Chao Gao, and Thomas Kirkland are employees of Promega Corporation. Promega develops and commercializes bioluminescence imaging technologies and reagents related to NanoLuc, HaloTag, Nano-Glo, FFz, and the reporter systems evaluated in this study. The remaining authors declare no competing financial interests.

## Supplementary Material

CB-OLF-D6CB00175K-s001

CB-OLF-D6CB00175K-s002

CB-OLF-D6CB00175K-s003

CB-OLF-D6CB00175K-s004

## Data Availability

The data supporting this article (supplementary figures and primer table) have been included as part of the supplementary information. Supplementary information (SI) is available. See DOI: https://doi.org/10.1039/d6cb00175k. Data for this article, including raw data of the graphs, is available at Zenodo at https://doi.org/10.5281/zenodo.22661680.

## References

[cit1] Prescher J. A., Contag C. H. (2010). Guided by the light: visualizing biomolecular processes in living animals with bioluminescence. Curr. Opin. Chem. Biol..

[cit2] Mezzanotte L., van’t Root M., Karatas H., Goun E. A., Löwik C. W. G. M. (2017). In Vivo Molecular Bioluminescence Imaging: New Tools and Applications. Trends Biotechnol..

[cit3] Hall M. P., Unch J., Binkowski B. F., Valley M. P., Butler B. L., Wood M. G., Otto P., Zimmerman K., Vidugiris G., Machleidt T., Robers M. B., Benink H. A., Eggers C. T., Slater M. R., Meisenheimer P. L., Klaubert D. H., Fan F., Encell L. P., Wood K. V. (2012). Engineered luciferase reporter from a deep sea shrimp utilizing a novel imidazopyrazinone substrate. ACS Chem. Biol..

[cit4] Su Y., Walker J. R., Park Y., Smith T. P., Liu L. X., Hall M. P., Labanieh L., Hurst R., Wang D. C., Encell L. P., Kim N., Zhang F., Kay M. A., Casey K. M., Majzner R. G., Cochran J. R., Mackall C. L., Kirkland T. A., Lin M. Z. (2020). Novel NanoLuc substrates enable bright two-population bioluminescence imaging in animals. Nat. Methods.

[cit5] Zambito G., Chawda C., Mezzanotte L. (2021). Emerging tools for bioluminescence imaging. Curr. Opin. Chem. Biol..

[cit6] Jacques S. L. (2013). Optical properties of biological tissues: a review. Phys. Med. Biol..

[cit7] Hemmer E., Benayas A., Légaré F., Vetrone F. (2016). Exploiting the biological windows: current perspectives on fluorescent bioprobes emitting above 1000 nm. Nanoscale Horiz..

[cit8] Xu Y., Piston D. W., Johnson C. H. (1999). A bioluminescence resonance energy transfer (BRET) system: application to interacting circadian clock proteins. Proc. Natl. Acad. Sci. U. S. A..

[cit9] Dale N. C., Johnstone E. K. M., White C. W., Pfleger K. D. G. (2019). NanoBRET: The Bright Future of Proximity-Based Assays. Front. Bioeng. Biotechnol..

[cit10] De A., Ray P., Loening A. M., Gambhir S. S. (2009). BRET3: a red-shifted bioluminescence resonance energy transfer (BRET)-based integrated platform for imaging protein-protein interactions from single live cells and living animals. FASEB J..

[cit11] Dragulescu-Andrasi A., Chan C. T., De A., Massoud T. F., Gambhir S. S. (2011). Bioluminescence resonance energy transfer (BRET) imaging of protein–protein interactions within deep tissues of living subjects. Proc. Natl. Acad. Sci. U. S. A..

[cit12] Saito K., Chang Y.-F., Horikawa K., Hatsugai N., Higuchi Y., Hashida M., Yoshida Y., Matsuda T., Arai Y., Nagai T. (2012). Luminescent proteins for high-speed single-cell and whole-body imaging. Nat. Commun..

[cit13] Rumyantsev K. A., Turoverov K. K., Verkhusha V. V. (2016). Near-infrared bioluminescent proteins for two-color multimodal imaging. Sci. Rep..

[cit14] Nishihara R., Paulmurugan R., Nakajima T., Yamamoto E., Natarajan A., Afjei R., Hiruta Y., Iwasawa N., Nishiyama S., Citterio D., Sato M., Kim S. B., Suzuki K. (2019). Highly bright and stable NIR-BRET with blue-shifted coelenterazine derivatives for deep-tissue imaging of molecular events in vivo. Theranostics.

[cit15] Machleidt T., Woodroofe C. C., Schwinn M. K., Méndez J., Robers M. B., Zimmerman K., Otto P., Daniels D. L., Kirkland T. A., Wood K. V. (2015). NanoBRET--A Novel BRET Platform for the Analysis of Protein-Protein Interactions. ACS Chem. Biol..

[cit16] Chu J., Oh Y., Sens A., Ataie N., Dana H., Macklin J. J., Laviv T., Welf E. S., Dean K. M., Zhang F., Kim B. B., Tang C. T., Hu M., Baird M. A., Davidson M. W., Kay M. A., Fiolka R., Yasuda R., Kim D. S., Ng H.-L., Lin M. Z. (2016). A bright cyan-excitable orange fluorescent protein facilitates dual-emission microscopy and enhances bioluminescence imaging *in vivo*. Nat. Biotechnol..

[cit17] Yeh H.-W., Karmach O., Ji A., Carter D., Martins-Green M. M., Ai H.-W. (2017). Red-shifted luciferase-luciferin pairs for enhanced bioluminescence imaging. Nat. Methods.

[cit18] Hattori M., Wazawa T., Orioka M., Hiruta Y., Nagai T. (2025). Creating coveted bioluminescence colors for simultaneous multi-color bioimaging. Sci. Adv..

[cit19] Suzuki K., Kimura T., Shinoda H., Bai G., Daniels M. J., Arai Y., Nakano M., Nagai T. (2016). Five colour variants of bright luminescent protein for real-time multicolour bioimaging. Nat. Commun..

[cit20] Los G. V., Encell L. P., McDougall M. G., Hartzell D. D., Karassina N., Zimprich C., Wood M. G., Learish R., Ohana R. F., Urh M., Simpson D., Mendez J., Zimmerman K., Otto P., Vidugiris G., Zhu J., Darzins A., Klaubert D. H., Bulleit R. F., Wood K. V. (2008). HaloTag: A Novel Protein Labeling Technology for Cell Imaging and Protein Analysis. ACS Chem. Biol..

[cit21] Farrants H., Shuai Y., Lemon W. C., Monroy Hernandez C., Zhang D., Yang S., Patel R., Qiao G., Frei M. S., Plutkis S. E., Grimm J. B., Hanson T. L., Tomaska F., Turner G. C., Stringer C., Keller P. J., Beyene A. G., Chen Y., Liang Y., Lavis L. D., Schreiter E. R. (2024). A modular chemigenetic calcium indicator for multiplexed in vivo functional imaging. Nat. Methods.

[cit22] Mohar B., Michel G., Wang Y.-Z., Hernandez V., Grimm J. B., Park J.-Y., Patel R., Clarke M., Brown T. A., Bergmann C., Gebis K. K., Wilen A. P., Liu B., Johnson R., Graves A., Tchumatchenko T., Savas J. N., Fornasiero E. F., Huganir R. L., Tillberg P. W., Lavis L. D., Svoboda K., Spruston N. (2025). DELTA: a method for brain-wide measurement of synaptic protein turnover reveals localized plasticity during learning. Nat. Neurosci..

[cit23] Grimm J. B., Muthusamy A. K., Liang Y., Brown T. A., Lemon W. C., Patel R., Lu R., Macklin J. J., Keller P. J., Ji N., Lavis L. D. (2017). A general method to fine-tune fluorophores for live-cell and in vivo imaging. Nat. Methods.

[cit24] Grimm J. B., English B. P., Chen J., Slaughter J. P., Zhang Z., Revyakin A., Patel R., Macklin J. J., Normanno D., Singer R. H., Lionnet T., Lavis L. D. (2015). A general method to improve fluorophores for live-cell and single-molecule microscopy. Nat. Methods.

[cit25] Grimm J. B., Tkachuk A. N., Xie L., Choi H., Mohar B., Falco N., Schaefer K., Patel R., Zheng Q., Liu Z., Lippincott-Schwartz J., Brown T. A., Lavis L. D. (2020). A general method to optimize and functionalize red-shifted rhodamine dyes. Nat. Methods.

[cit26] Porter K., Killoran M., Hurst R., Klein M., De Bakshi D., Esmatpour R., Encell L. P., Gama-Franceschi F., Bessetti J., Riching K. M., Machleidt T., Mezzanotte L., Friedman Ohana R. (2026). A Modular Platform for Quantitative Two-Color Bioluminescence Combining NanoLuc and Red-Shifted NanoPrism Luciferases. ACS Chem. Biol..

[cit27] Hiblot J., Yu Q., Sabbadini M. D. B., Reymond L., Xue L., Schena A., Sallin O., Hill N., Griss R., Johnsson K. (2017). Luciferases with Tunable Emission Wavelengths. Angew. Chem., Int. Ed..

[cit28] Zheng Q., Ayala A. X., Chung I., Weigel A. V., Ranjan A., Falco N., Grimm J. B., Tkachuk A. N., Wu C., Lippincott-Schwartz J., Singer R. H., Lavis L. D. (2019). Rational Design of Fluorogenic and Spontaneously Blinking Labels for Super-Resolution Imaging. ACS Cent. Sci..

[cit29] Kuchimaru T., Iwano S., Kiyama M., Mitsumata S., Kadonosono T., Niwa H., Maki S., Kizaka-Kondoh S. (2016). A luciferin analogue generating near-infrared bioluminescence achieves highly sensitive deep-tissue imaging. Nat. Commun..

[cit30] Iwano S., Sugiyama M., Hama H., Watakabe A., Hasegawa N., Kuchimaru T., Tanaka K. Z., Takahashi M., Ishida Y., Hata J., Shimozono S., Namiki K., Fukano T., Kiyama M., Okano H., Kizaka-Kondoh S., McHugh T. J., Yamamori T., Hioki H., Maki S., Miyawaki A. (2018). Single-cell bioluminescence imaging of deep tissue in freely moving animals. Science.

[cit31] Saito-Moriya R. , Iwano S., Kitada N., Yamasaki N., Kamiya G., Ogo K., Sugiyama T., Chuluun-Erdene A., Isagawa T., Obata R., Ijuin R., Abe H., Kashiwakura Y., Mabuchi Y., Takahashi M., Yamamoto Y., Sawaki D., Sato T., Sato S., Nishikii H., Yoshimura K., Hioki H., Ohmori T., Nakashiba T., Hirano T., Aoyama H., Takeda N., Miyawaki A., Maki S. A. and Kuchimaru T., A bright synthetic near-infrared luciferin enhances the capabilities of deep-tissue bioluminescence imaging using firefly luciferases, bioRxiv, 2025, preprint 10.1101/2025.01.20.633992

[cit32] Stowe C. L., Burley T. A., Allan H., Vinci M., Kramer-Marek G., Ciobota D. M., Parkinson G. N., Southworth T. L., Agliardi G., Hotblack A., Lythgoe M. F., Branchini B. R., Kalber T. L., Anderson J. C., Pule M. A. (2019). Near-infrared dual bioluminescence imaging in mouse models of cancer using infraluciferin. eLife.

[cit33] Zambito G., Hall M. P., Wood M. G., Gaspar N., Ridwan Y., Stellari F. F., Shi C., Kirkland T. A., Encell L. P., Löwik C., Mezzanotte L. (2021). Red-shifted click beetle luciferase mutant expands the multicolor bioluminescent palette for deep tissue imaging. iScience.

[cit34] Zambito G., Mezzanotte L. (2021). Near-infrared bioluminescence imaging of two cell populations in living mice. STAR Protoc..

[cit35] Chen Z., Jiang L., Liu X., Shi Y., Liu Y., Jiang L., Li J., Huang X., Zhou S., Bao B., Zhang B., Lin Q., Bian Q., Yin Y., Zuo F., Su N., Tang Y., Zhao Y., Yang Y., Chen X., Zhu L. (2026). NirFAP680: a highly bright and stable large Stokes shift near-infrared fluorogen-activating protein. Nat. Methods.

[cit36] Branchini B. R., Southworth T. L., Khattak N. F., Michelini E., Roda A. (2005). Red- and green-emitting firefly luciferase mutants for bioluminescent reporter applications. Anal. Biochem..

[cit37] Brennan C. K., Yao Z., Ionkina A. A., Rathbun C. M., Sathishkumar B., Prescher J. A. (2022). Multiplexed bioluminescence imaging with a substrate unmixing platform. Cell Chem. Biol..

[cit38] Maranesi A., Mohammadi S., Castañon I., Gama-Franceschi F., Falciani C., Pini A., Mezzanotte L., Unger W., Ferrari A. (2025). The identity of implant materials governs the antimicrobial efficacy of SET-M33. Sci. Rep..

