# Supplementary material for: PrismaLuc luciferase enables tunable near-infrared bioluminescence for in vivo imaging
Source: RSC Chem Biol. 2026 Sep 7;7(11):2188–99. doi: 10.1039/d6cb00175k (PMC13565418; doi:10.1039/d6cb00175k)

Supplementary Information (SI) for RSC Chemical Biology.
This journal is © The Royal Society of Chemistry 2026

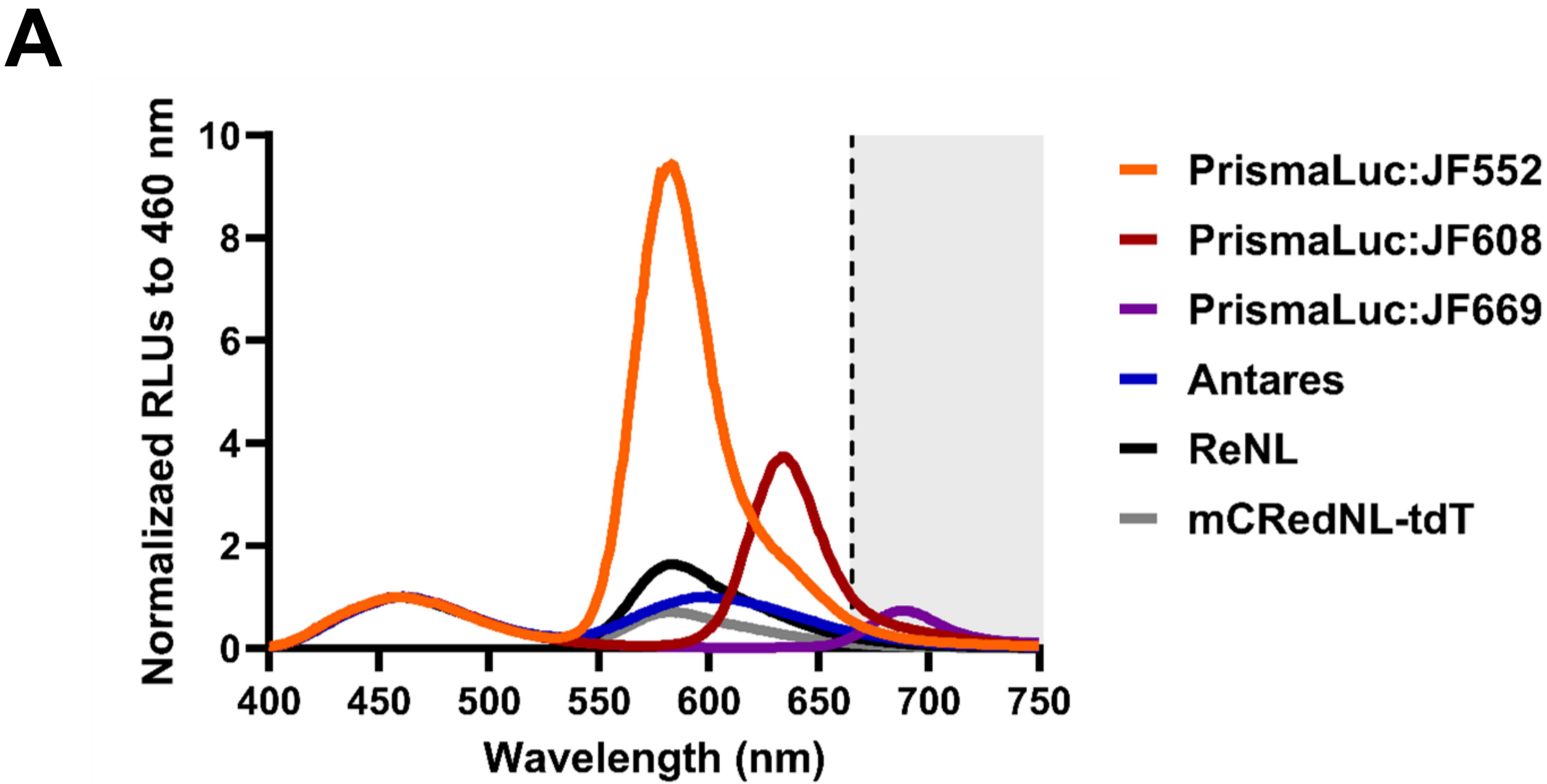

Supplement: Supplementary file 1 [file CB-007-D6CB00175K-s001.pdf]
