# Supplementary material for: PrismaLuc luciferase enables tunable near-infrared bioluminescence for in vivo imaging
Source: RSC Chem Biol. 2026 Sep 7;7(11):2188–99. doi: 10.1039/d6cb00175k (PMC13565418; doi:10.1039/d6cb00175k)
Supplement: Supplementary file 4 [file CB-007-D6CB00175K-s004.pdf]

This journal is © The Royal Society of Chemistry 2026

Supplemental Material

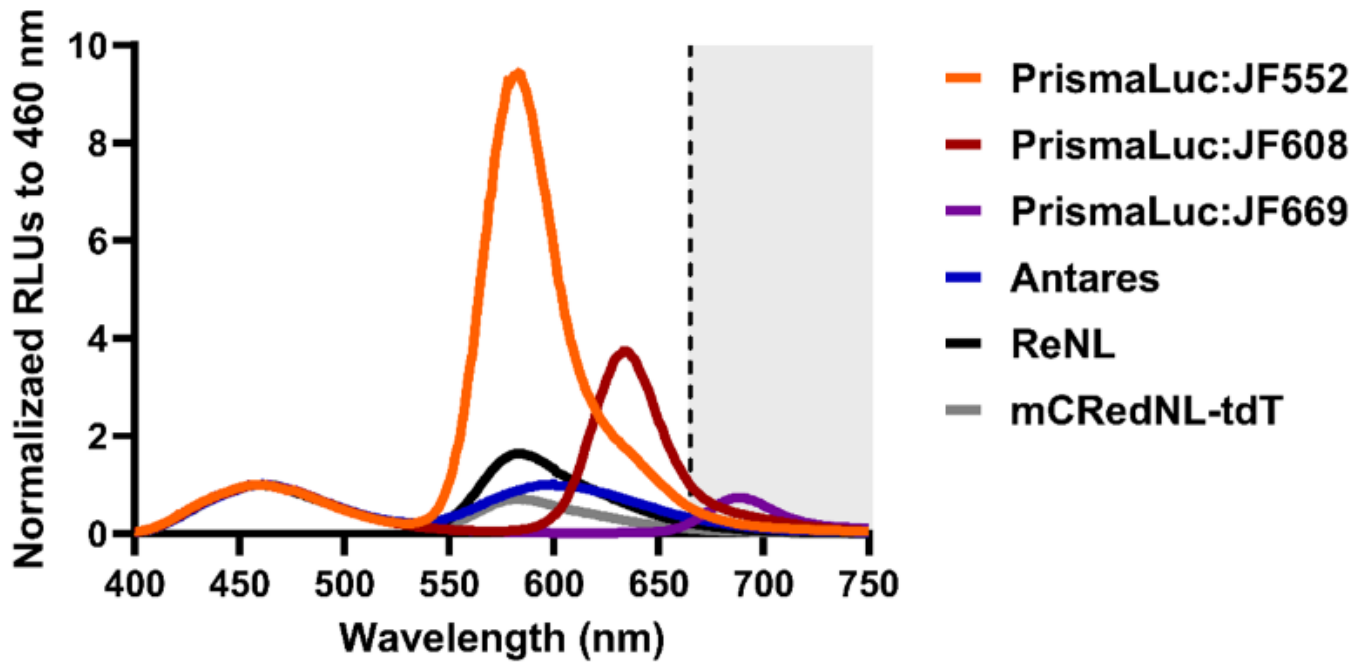

Fig. S1 Spectral comparison of PrismaLuc and established BRET systems in HCT116 cells. Normalized spectra to the 460 nm donor peak of HCT116 cells expressing PrismaLuc, Antares, or NanoLantern following mRNA transfection and substrate addition. The shaded region indicates wavelengths above 665 nm.

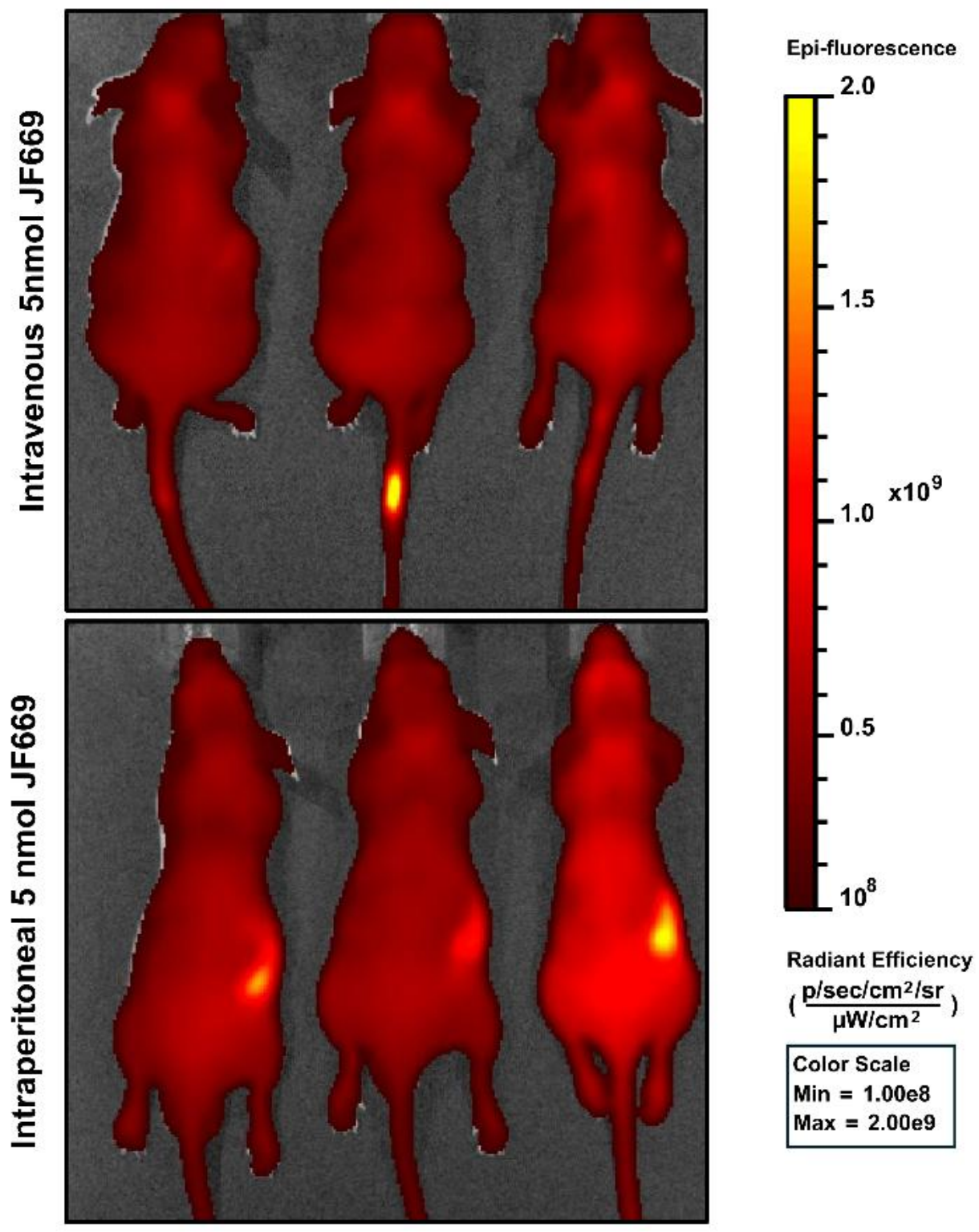

Fig. S2 In vivo bioavailability of 5 nmol JF669 HaloTag ligand. Whole-body fluorescence imaging of BALB/c nude mice following intraperitoneal or intravenous administration of 5 nmol of JF669 HaloTag ligand. Fluorescence signal was detected 1 minute after injection.

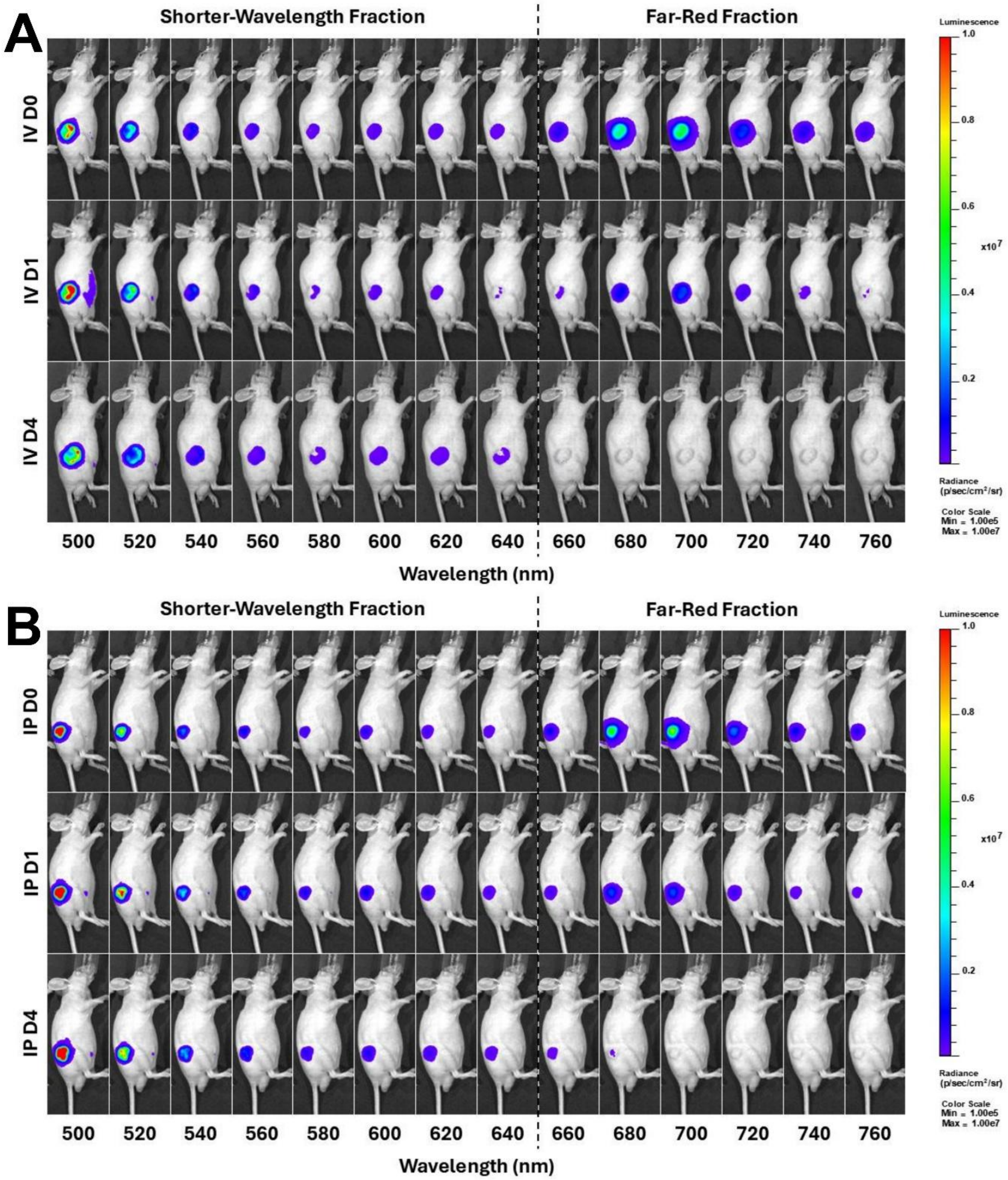

Fig. S3 Longitudinal spectral imaging of PrismaLuc:JF669 tumours after IV or IP JF669 administration. Representative full spectral image series of BALB/c nude mice bearing HCT116-PrismaLuc tumours after intravenous (A) or intraperitoneal (B) administration of JF669 HaloTag ligand. Images were acquired at Day 0, Day 1, and Day 4 following FFz substrate administration using sequential 20 nm band-pass filters from 500 to 760 nm. Images are separated into shorter-wavelength and far-red fractions limited by the IVIS filters, with the dashed line indicating the transition between 640 and 660 nm.

Primer Table

Plasmid	Primer Name	Sequence (5' – 3')
JPNLrT1	PrismaLuc Fow	CCGACTAAGGAGGTATGTATGAAGCATCACCACCATCATCAC
	PrismaLuc Rev	TCGTTTTATTTGATGCCTGGTTAGCCGGAAATCTCGAGCGTC
	BB PNL Fow	CGCTCGAGATTTCCGGCTAACCAGGCATCAAATAAAACGAAAGG
	BB PNL Rev	GATGATGGTGGTGATGCTTCATACATACCTCCTTAGTCGGCGC
