# Supplementary material for: PrismaLuc luciferase enables tunable near-infrared bioluminescence for in vivo imaging
Source: RSC Chem Biol. 2026 Sep 7. Online ahead of print. doi: 10.1039/d6cb00175k (PMC13565418; doi:10.1039/d6cb00175k)
Supplement: Supplementary file 4 [file CB-OLF-D6CB00175K-s004.pdf]

This journal is © The Royal Society of Chemistry 2026



**Supplemental Material**

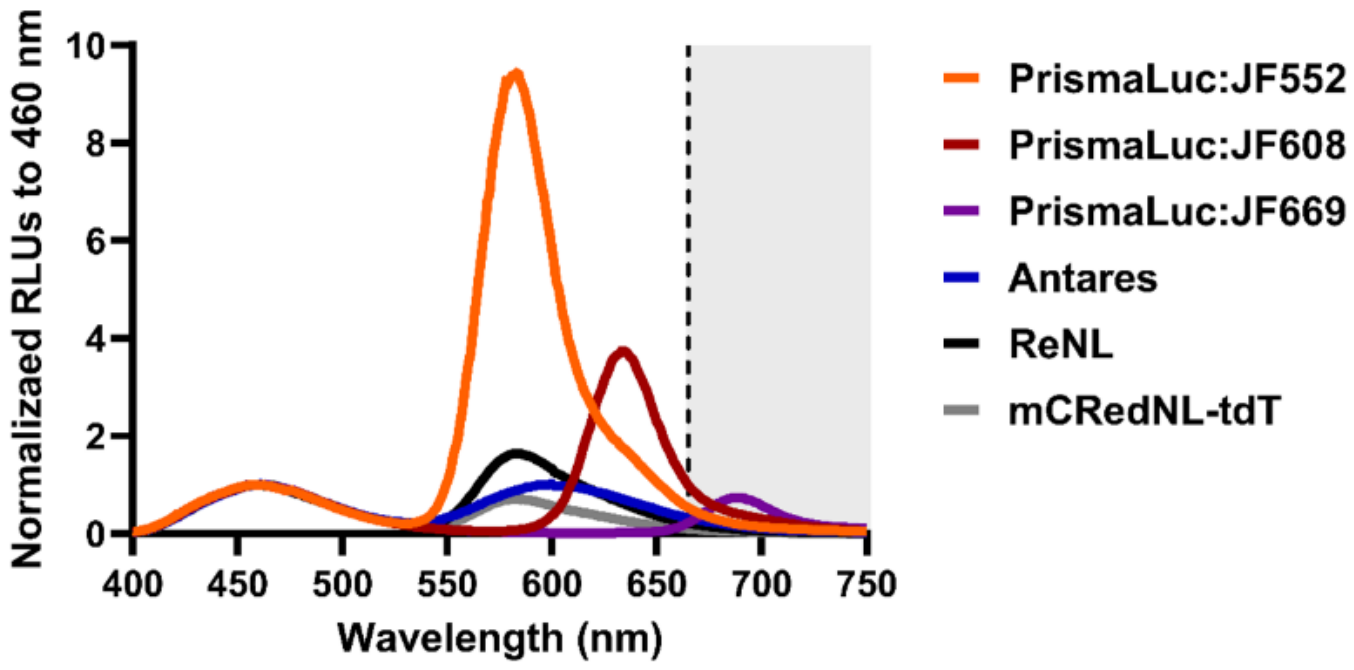


**Fig. S1 Spectral comparison of PrismaLuc and established BRET systems in HCT116 cells.** Normalized spectra to the 460 nm donor peak of HCT116 cells expressing PrismaLuc, Antares, or NanoLantern following mRNA transfection and substrate addition. The shaded region indicates wavelengths above 665 nm.

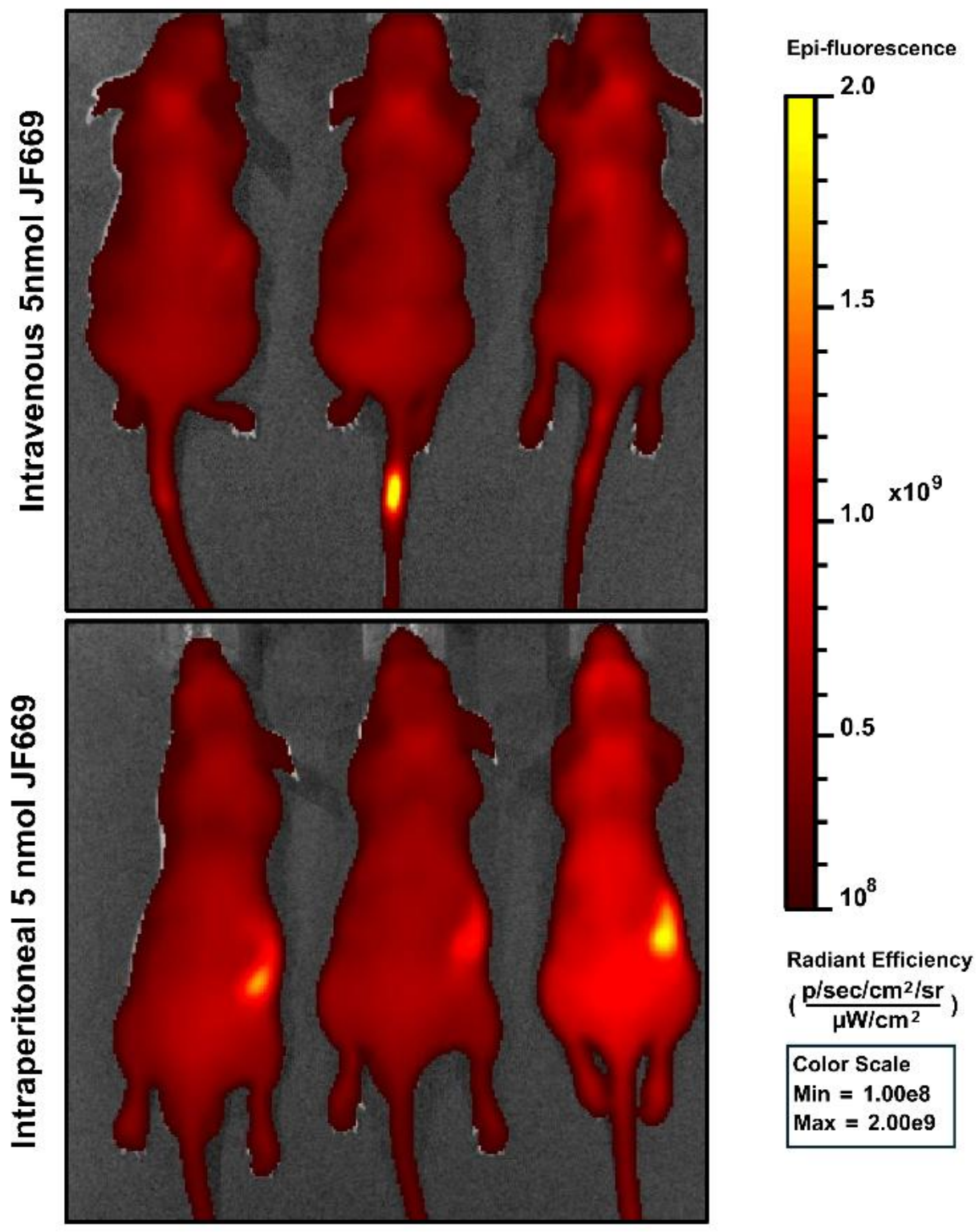


**Fig. S2 In vivo bioavailability of 5 nmol JF669 HaloTag ligand.** Whole-body fluorescence imaging of BALB/c nude mice following intraperitoneal or intravenous administration of 5 nmol of JF669 HaloTag ligand. Fluorescence signal was detected 1 minute after injection.

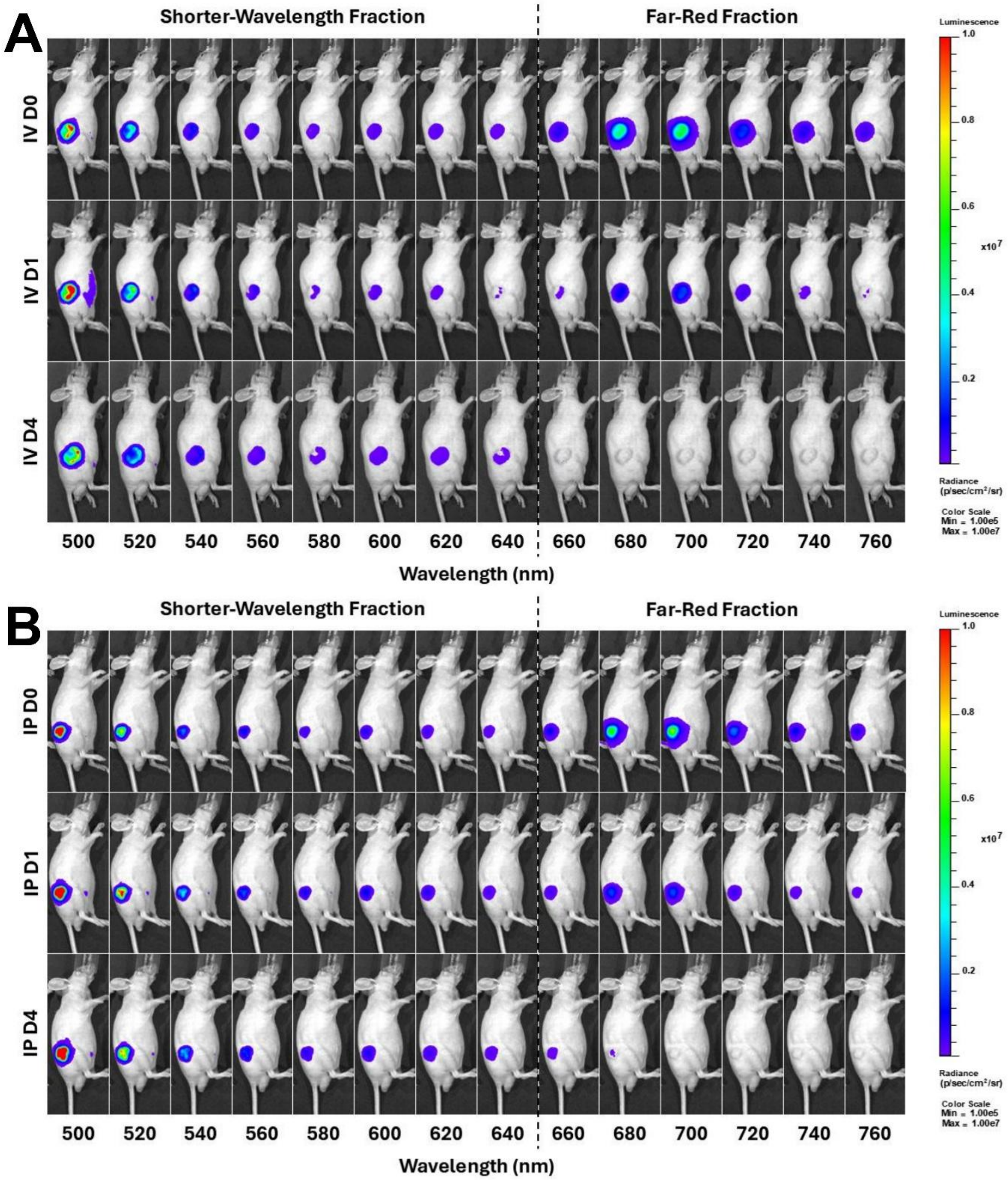


**Fig. S3 Longitudinal spectral imaging of PrismaLuc:JF669 tumours after IV or IP JF669 administration.** Representative full spectral image series of BALB/c nude mice bearing HCT116-PrismaLuc tumours after intravenous (A) or intraperitoneal (B) administration of JF669 HaloTag ligand. Images were acquired at Day 0, Day 1, and Day 4 following FFz substrate administration using sequential 20 nm band-pass filters from 500 to 760 nm. Images are separated into shorter-wavelength and far-red fractions limited by the IVIS filters, with the dashed line indicating the transition between 640 and 660 nm.

## Primer Table

| Plasmid | Primer Name | Sequence (5' – 3') |
|---|---|---|
| JPNLrT1 | PrismaLuc Fow | CCGACTAAGGAGGTATGTATGAAGCATCACCACCATCATCAC |
| | PrismaLuc Rev | TCGTTTTATTTGATGCCTGGTTAGCCGGAAATCTCGAGCGTC |
| | BB PNL Fow | CGCTCGAGATTTCCGGCTAACCAGGCATCAAATAAAACGAAAGG |
| | BB PNL Rev | GATGATGGTGGTGATGCTTCATACATACCTCCTTAGTCGGCGC |
